# Attention 3D UNET for dose distribution prediction of high‐dose‐rate brachytherapy of cervical cancer: Intracavitary applicators

**DOI:** 10.1002/acm2.14568

**Published:** 2024-11-15

**Authors:** Suman Gautam, Alexander F. I. Osman, Dylan Richeson, Somayeh Gholami, Binod Manandhar, Sharmin Alam, William Y. Song

**Affiliations:** ^1^ Department of Radiation Oncology Virginia Commonwealth University Richmond Virginia USA; ^2^ Department of Radiation Oncology Inova Schar Cancer Institute Fairfax Virginia USA; ^3^ Department of Radiation Oncology University of Utah Salt Lake City Utah USA; ^4^ Department of Radiation Oncology Baylor Scott and White Health Temple Texas USA

**Keywords:** attention‐gated UNET, brachytherapy, cervical cancer, deep‐learning, dose prediction

## Abstract

**Background:**

Formulating a clinically acceptable plan within the time‐constrained clinical setting of brachytherapy poses challenges to clinicians. Deep learning based dose prediction methods have shown favorable solutions for enhancing efficiency, but development has primarily been on external beam radiation therapy. Thus, there is a need for translation to brachytherapy.

**Purpose:**

This study proposes a dose prediction model utilizing an attention‐gating mechanism and a 3D UNET for cervical cancer high‐dose‐rate intracavitary brachytherapy treatment planning with tandem‐and‐ovoid/ring applicators.

**Methods:**

A multi‐institutional data set consisting of 77 retrospective clinical brachytherapy plans was utilized in this study. The data were preprocessed and augmented to increase the number of plans to 252. A 3D UNET architecture with attention gates was constructed and trained for mapping the contour information to dose distribution. The trained model was evaluated on a testing data set using various metrics, including dose statistics and dose‐volume indices. We also trained a baseline UNET model for a fair comparison.

**Results:**

The attention‐gated 3D UNET model exhibited competitive accuracy in predicting dose distributions similar to the ground truth. The average values of the mean absolute errors were 0.46 ± 11.71 Gy (vs. 0.47 ± 9.16 Gy for a baseline UNET) in CTV_HR_, 0.55 ± 0.67 Gy (vs. 0.70 ± 1.54 Gy for a baseline UNET) in bladder, 0.42 ± 0.46 Gy (vs. 0.49 ± 1.34 Gy for a baseline UNET) in rectum, and 0.31 ± 0.65 Gy (vs. 0.20 ± 3.76 Gy for a baseline UNET) in sigmoid. Our results showed that the mean individual differences in ΔD_2cc_ for bladder, rectum, and sigmoid were 0.38 ± 1.19 (*p* = 0.50), 0.43 ± 0.71 (*p* = 0.41), and −0.47 ± 0.79 (*p* = 0.30) Gy, respectively. Similarly, the mean individual differences in ΔD_1cc_ for bladder, rectum, and sigmoid were 0.09 ± 1.21 (*p* = 0.36), 0.20 ± 0.95 (*p* = 0.24), and −0.21 ± 0.59 (*p* = 0.30) Gy. The mean individual differences for ΔD_90_, ΔV_100%_, ΔV_150%_, and ΔV_200%_ of the CTV_HR_ were −0.45 ± 2.42 (*p* = 0.26) Gy, 0.55 ± 9.42% (*p* = 0.78), 0.82 ± 4.21% (*p* = 0.81), and −0.80 ± 10.48% (*p* = 0.36), respectively. The model requires less than 5 s to predict a full 3D dose distribution for a new patient plan.

**Conclusion:**

Attention‐gated 3D UNET revealed a promising capability in predicting voxel‐wise dose distributions compared to 3D UNET. This model could be deployed for clinical use to predict 3D dose distributions for near real‐time decision‐making before planning, quality assurance, and guiding future automated planning, making the current workflow more efficient.

## 1 INTRODUCTION

Cervical cancer is ranked as the fourth most prevalent cancer among women in the United States, leading to an estimated mortality of 14 100 new cases in 2022.^1^ High‐dose‐rate brachytherapy (HDR‐BT) is a significant therapeutic approach utilized either as a monotherapy or in combination with external beam radiotherapy (EBRT).^2^ The fundamental principle in radiotherapy is to deliver the prescribed dose to the intended target while sparing the critical organs at risk (OARs). Within HDR‐BT, the radioactive source is positioned inside or near the tumor sites using applicators introduced into the vaginal cavity, such as tandem‐and‐ovoids (T&O) or tandem‐and‐rings (T&R). A notable advantage of HDR‐BT lies in its ability to concentrate a potent radiation dose onto the intended target through applicators, resulting in a focused radiation dose to the target while minimizing exposure to OARs.

Creating a plan that meets clinically acceptable standards in BT is a non‐trivial task. It involves iterative manual adjustments driven by several factors such as applicator selection, patient‐specific anatomy, and conflicting optimization constraints. Clinical factors such as comorbidities or tumor pathology/radiobiology also play a role in this process. Nevertheless, the current image‐guided‐adaptive brachytherapy (IGABT) treatment workflow, which often involves inverse planning optimization, demands swift completion of the planning process when operating under tight time constraints.^3^ Therefore, this raises the need for a more time‐efficient approach to creating a treatment plan to avoid patient movement‐induced applicator dislocation. The other challenge is the standardization of plan quality, as the effectiveness of BT is highly dependent on physician‐skill and technique.^4^ As a result, the time‐intensive nature of this procedure, combined with the significant variations among physicians/planners, has served as a driving force for researchers to introduce automation. This automated process can help establish uniformity/standardized practices across clinics and maintain high‐quality planning. A recent study^5^ has indicated that plans derived from traditional inverse planning methods might not guarantee the stability and reliability of plan quality. This is due to the considerable challenges encountered in solving analytical formulas using traditional dose planning methods. These methods rely on solving nonlinear mathematical models, which might not be as consistent as previously thought. Therefore, significant advancement of deep learning at this point can augment the shortfalls in inverse planning algorithms and the optimization processes.

Several methods have been suggested to enhance the efficiency and quality of EBRT and BT treatment planning workflows.^6^ Advancements in EBRT include the development of novel techniques like intensity‐modulated radiation therapy, volumetric‐modulated arc therapy,^7^, ^8^ and station parameter‐optimized radiation therapy, including knowledge‐based planning (KBP)^9^, ^10^ and, more recently, deep learning (DL)‐based methods.^11^, ^12^ KBP assumes a geometrical relationship between the target and OARs for every patient. These geometrical relationships correlate with dose‐by‐overlap volume histograms and distance‐to‐target histograms.^13^

DL provides several benefits throughout the various stages of radiotherapy treatment planning. One of its strengths is its ability to extract features from the raw dataset to build the predictive model. This predictive model is subsequently employed to predict the dose distribution for a new patient. The outcomes of the predicted dosimetric results can be utilized in the plan optimization process to enhance the quality of the plan. UNET is a widely used DL network known for its success in dose prediction, attributed to its simple model structures and its ability to effectively retain low‐level features.^14^ Several studies have used 2D/3D UNET^15^ and its variants^16^ generated from UNET, including 3D dense UNET^17^, ^18^ and attention UNET^18^, ^19^ to estimate dose distributions. Osman et al.^20^, ^21^ utilized an attention UNET for 3D dose prediction of head‐and‐neck cancer to identify the most informative features to predict the dose distribution. Ming et al.^22^ used a 3D deep convolutional neural network (CNN) for the dose distribution prediction for cervical cancer BT.

This study introduces a rapid and automated tool for estimating dose distributions in HDR‐BT T&O/T&R planning. Attention‐gated 3D UNET was developed and integrated into conventional CNN models with minimal computational impact while increasing the model prediction accuracy. We employed UNET as a baseline model because it has a simple architecture, is extensively applied for dose prediction in EBRT, and has demonstrated efficacy in dose prediction.^18^, ^20^, ^23^ The strength of integrating an attention‐gated mechanism to UNET allows the convolutional network to focus on highlighting the salient features of the sequence while suppressing the irrelevant counterparts, making the prediction more contextualized. This could be beneficial for BT where there are numerous OARs adjacent to the target in addition to the sharp dose gradient nature of BT in the region outside the target. Thus, the attention mechanism could aid the model in better discriminating the surrounding tissue structures of tumors, leading to a more precise prediction of dose distribution.

## 2 MATERIALS AND METHODS

### 2.1 Clinical datasets

Institutional Review Board approved, clinically delivered treatment plans from 37 cervical cancer patients treated with computed tomography (CT)‐guided T&O or T&R HDR‐BT from three institutions were included in this study. In total, 73 treatment plans, each patient consisting of two to four fractions, were included in this study.^12^, ^24^, ^25^ The data were initially divided into a development (80% of the plans, *n* = 59) and a testing set (20%, *n* = 14). The model's ability and stability in predicting new cases were evaluated using a testing cohort set of 14 treatment plans. DL models rely on big data to avoid overfitting.^23^ Data augmentation^26^ was applied to the images (contour masks) as well as the dose distribution on the development set (*n* = 59), and this was limited to rotation and translations (diagonal shift, width shift, height shift)^27^ to ensure the dose calculation remains valid (e.g., avoiding stretches or shifts in applicators relative to patient anatomy). This process would help to minimize the likelihood of model overfitting due to limited data. The transformation increased the number of plans four times (*n* = 236). The development set after applying the augmentation (*n* = 236) was then split into a training set (80%, *n* = 189) and a validation set (20%, *n* = 47). The CT scans were acquired with a pixel size of 0.6 mm × 0.6 mm and a slice thickness of 3 mm, while the dose was calculated on each CT slice in the BrachyVision Treatment Planning System (BV‐TPS) using a grid size of 2.5 × 2.5 mm^2^. The contours, CT images, and dose distributions were up/down‐sampled (crop and resize) to 64 × 64 × 64 voxels with a spatial resolution of 1 × 1 mm^2^, while the z dimension remained constant at 3 mm. Therefore, all data had become in a good matching. Increasing the resolution further (higher than 1 × 1 × 3 mm^3^) might improve the prediction accuracy, particularly at the high‐dose gradient regions, but our computational memory does not permit handling more data.

### 2.2 Treatment planning

BrachyVision Treatment Planning System was used to optimize all the clinical plans. Prescription doses were in the range of 4.8–7.0 Gy/fraction. EQD2 (equivalent dose in 2‐Gy fractions) was calculated using α/β = 10 Gy for the target and α/β = 3 Gy for OAR, to allow accumulation of EBRT and BT doses. The plan quality assessment for locally advanced cervical cancer was evaluated per international guidelines outlined by the American Brachytherapy Society (ABS).^28^ The planning constraints included CTV_HR_ D_90 _> 85 Gy, bladder D_2cc _< 90 Gy, rectum D_2cc _< 75 Gy, and sigmoid D_2cc _< 75 Gy. The planning procedure began with T&O and T&R dwell times set to a standard *pear shape* loading pattern and simultaneously ensuring compliance with ABS dosimetric guidelines. Radiation oncologists and medical physicists either manually or inverse‐optimized the dwell times to achieve optimal CTV_HR_ coverage while minimizing dose to OARs.

### 2.3 Data preprocessing

For every patient, the input data was organized into a four‐channel tensor, with each channel corresponding to specific structures: (0) bladder, (1) CTV_HR_, (2) rectum, and (3) sigmoid. The DICOM‐RT data, including CT, RT‐STRUCT, and RT‐DOSE, for each treatment plan was extracted from BV‐TPS and implemented on a Keras API utilizing TensorFlow (v2.9.1) as the backend within a Python environment (v3.9.15).^29^ In Python, the contours in the RT‐STRUCT were transformed into images with binary masks. The data size was cropped and resized to 64 × 64 × 64 voxels to ensure a uniform matrix size for each plan. Cropping was performed by removing the peripheral rows and columns with null information along the x, y, and z axes to minimize unimportant background area (i.e., by including a region with anatomical information) and avoid GPU/CPU memory overflow. The resizing was performed using the nearest neighbor interpolation.

### 2.4 Network architecture

**FIGURE 1:**
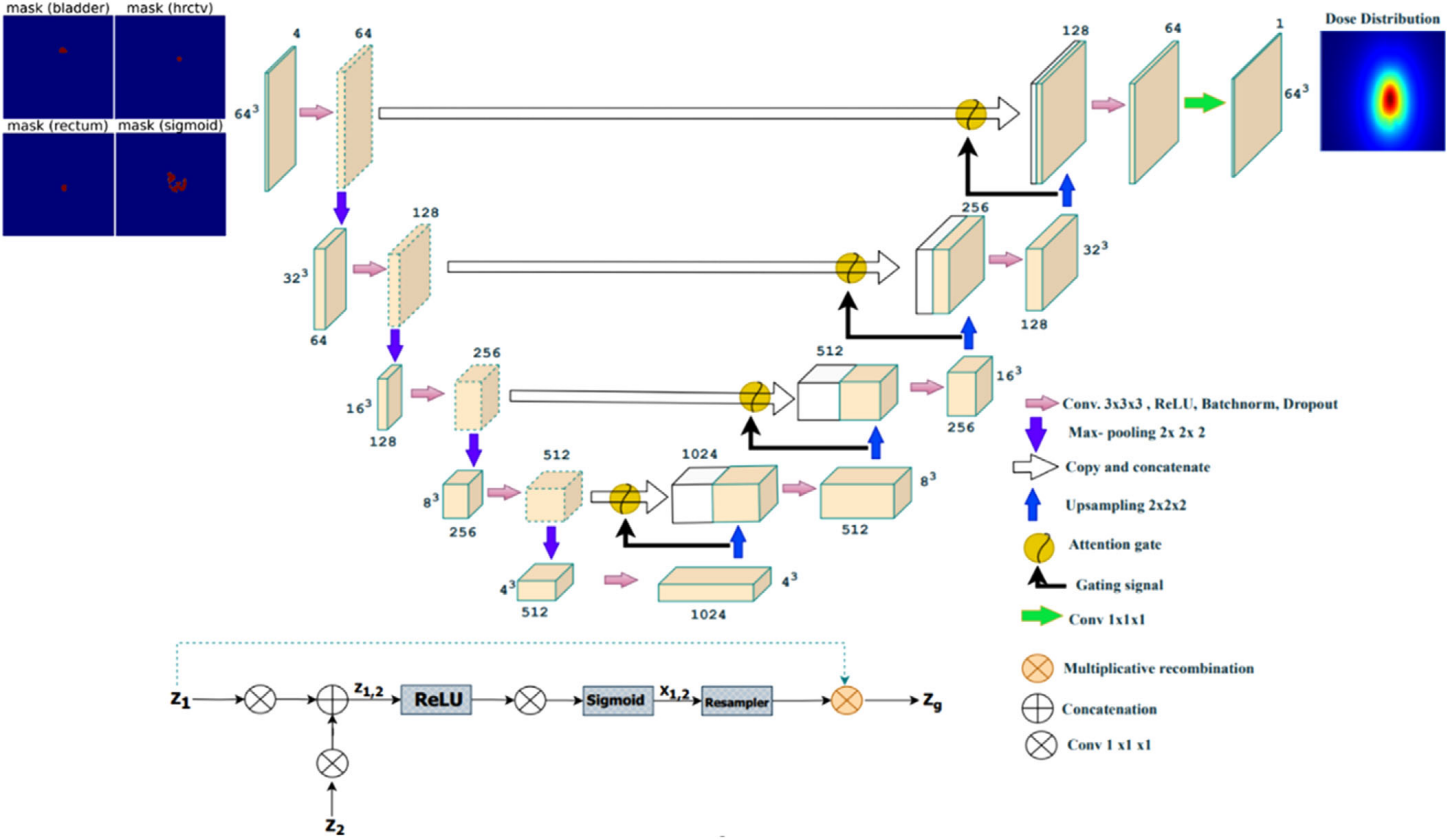
The attention‐gated 3D UNET architecture for brachytherapy dose prediction. The patient anatomical information is used as input to predict full 3D dose distribution. The number of extracted feature maps is denoted on the top/bottom of the cubes. The size of the feature maps is provided on the left/right side of the box. Yellow boxes correspond to a set of feature maps. White boxes represent copied feature maps. The arrows represent different operations. The attention gating mechanism is shown in the figure for the propagation signal z_1_, gating signal z_2_, and final gated output signal z_g_ for the network.

A 3D UNET, created by expanding the 2D UNET,^14^ was integrated with an attention gate in the upsampling layer for dose distribution prediction. Figure 1 shows the architecture of the attention‐gated 3D UNET.

UNET consists of three modules: an encoder for downsampling, a skip connection, and a decoder for upsampling. The encoder part consists of two consecutive 3 × 3 × 3 convolution layers, each followed sequentially by a batch normalization layer,^14^ a rectified linear unit (ReLu) activation function,^30^ and a 2 × 2 × 2 max pooling layer for a down‐sampling process. It extracts the global features while reducing the spatial dimension. The skip connection modules integrate the global abstraction features and spatial features of equal dimensions. The same padding was added to ensure that the feature size remained constant. The dropout rate^31^ was employed following the second convolutional layer, which progressively increases from 10% to 30% at each stage. Between two levels, a 2 × 2 × 2 max‐pooling was implemented to reduce the dimensions of the feature progressively from 64^3^ to 4^3^, but the number of derived features was increased by a factor of 2 (64 to 1024) at each level.

The decoder layer is the mirror version of the encoder layer. It consists of four iterative blocks, each comprising two 3 × 3 × 3 convolution layers followed by a batch normalization, ReLu activation, and a 2 × 2 × 2 deconvolution layer to facilitate the up‐sampling process. The decoder layer successively restores the initial dimension of the input data. It also continues to learn non‐linear relationships within the input data to reconstruct the targeted output tensor having identical resolution as the input from the representation space in the network.

The output from each convolutional block in the encoder is concatenated to its corresponding in the decoder through attention‐gated connections. These connections not only merge the global abstraction features and spatial features of the same size but also empower the network to suppress irrelevant features while highlighting salient features useful for the given task propagating through the architecture. This operation eventually leads to the recovery of a clean dose distribution image. The attention gates implement a 3D convolution with a kernel size of 1 × 1 × 1 and a voxel stride of 1 to a propagation signal (z_1_) and a 3D convolution with a kernel size of 1 × 1 × 1 and a voxel stride of 2 to a gating signal (z_2_). The signals z_1_ and z_2_ undergo addition, resulting in a combined activation (z_1,2_), which is then subjected to ReLU activation before passing through a 1 × 1 × 1 convolutional kernel. This helps to modulate multi‐scale level feature response propagation throughout the network. The output is activated sigmoidally to form x_1,2_. The final gated output signal (z_g_) is obtained by multiplying z_1_ by x_1_,_2_. The final layer comprises a 3D convolutional layer with a kernel size of 1 × 1 × 1 followed by a linear activation function, providing a voxel‐wise continuous output matching the resolution of the input data. The output is a one‐channel 3D dose distribution matrix of 64 × 64 × 64 voxels.

All the training parameters were initialized before the training process. The method of initialization was He uniform given by He et al.^32^ because it selects the initial weights from the range of values and shows better performance for ReLU layers. The training process minimizes the voxel‐wise loss between the predicted (d_predict_) and the ground‐truth (d_ground‐truth_) dose distribution using the mean absolute error (MAE) loss function:

$$\begin{array}{cc} & \mathrm{Loss}\;\mathrm{function}\;(\mathrm{prediction},\;\mathrm{ground}-\mathrm{truth})\\ & \;=\frac{1}{n}\sum_{i=1}^{n}\left|d_{prediction}^{i}-d_{ground-truth}^{i}\right| \end{array}$$

where “”" represents the voxel index out of “n” total voxels. The Adam stochastic algorithm was used for training the model with β1 = 0.9, β2 = 0.999, decay = 0, and learning rate = 0.001.^33^ The model was trained for 250 epochs with a batch of eight samples. To prevent the likelihood of model overfitting, two regularization techniques, namely the dropout and early stopping methods, were implemented. During the training process, the performance of the model was continuously validated.

### 2.5 Evaluation

The model's performance and generalizability were assessed using a testing cohort of 20% of treatment plans. MAE metric was used to compare the predicted dose distributions to the ground‐truth dose distributions. The MAE is given by this equation:

$$\begin{array}{cc} & \mathrm{MAE}\;\left(\mathrm{predict},\;\mathrm{ground}-\mathrm{truth}\right)\\ & \;=\frac{1}{n}\sum_{i=1}^{n}\left|d_{predict}^{i}-d_{ground-truth}^{i}\right|, \end{array}$$

which measures the average absolute differences between the predicted and ground‐truth doses across “n” data points. Additionally, voxel‐wise difference maps were generated to show the differences between the predicted and ground‐truth dose distributions by computing ($d_{predict}^{i}$—$d_{clinical}^{i}$) at each voxel. This dose differences map shows the errors in the predicted dose distributions. DVH indices for target and OARs, for example, D_max_, D_90_, V_100_, D_2cc_, and D_1cc_ were computed to quantitatively assess the quality of the predicted plans compared to the ground‐truth plans.

## 3 RESULTS

**FIGURE 2:**
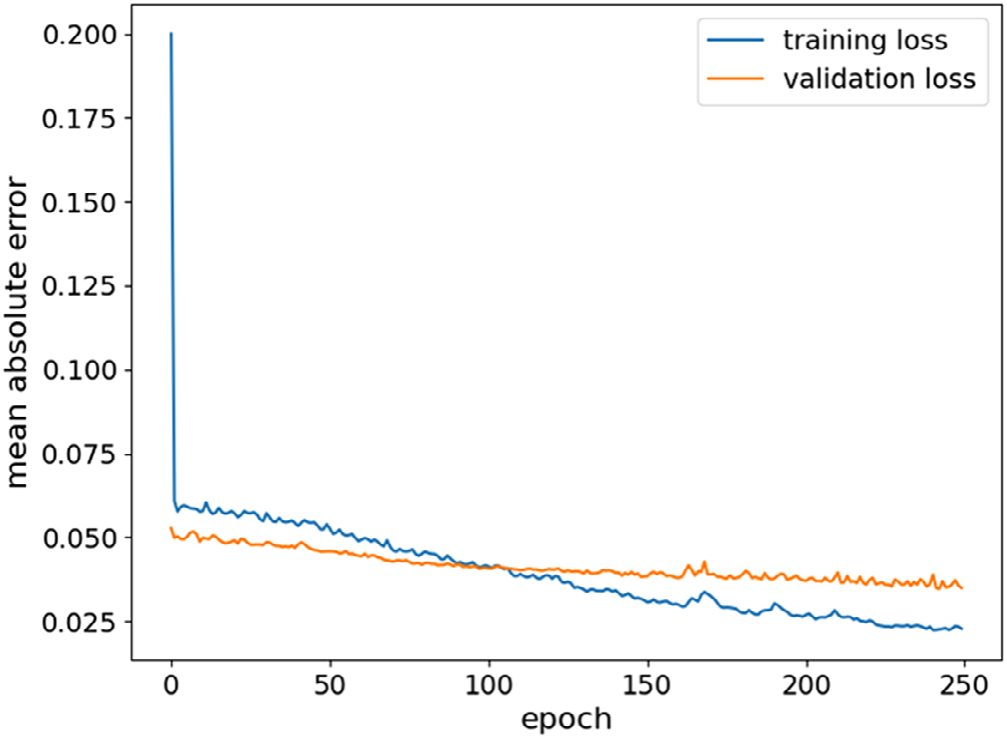
The learning curve of the training loss versus the validation loss over the number of epochs. This training curve highlights how the model is learning, and the validation curve highlights the model's generalizability.

Figure 2 illustrates the learning curve of the proposed model's performance, enabling us to determine whether the model has effectively learned the mapping. From the figure, there is no evidence of overfitting or underfitting. The training and validation losses are minimized consistently throughout the model training process until they reach convergence. The training and validation curves demonstrate similar performance with good generalization capabilities.

**FIGURE 3:**
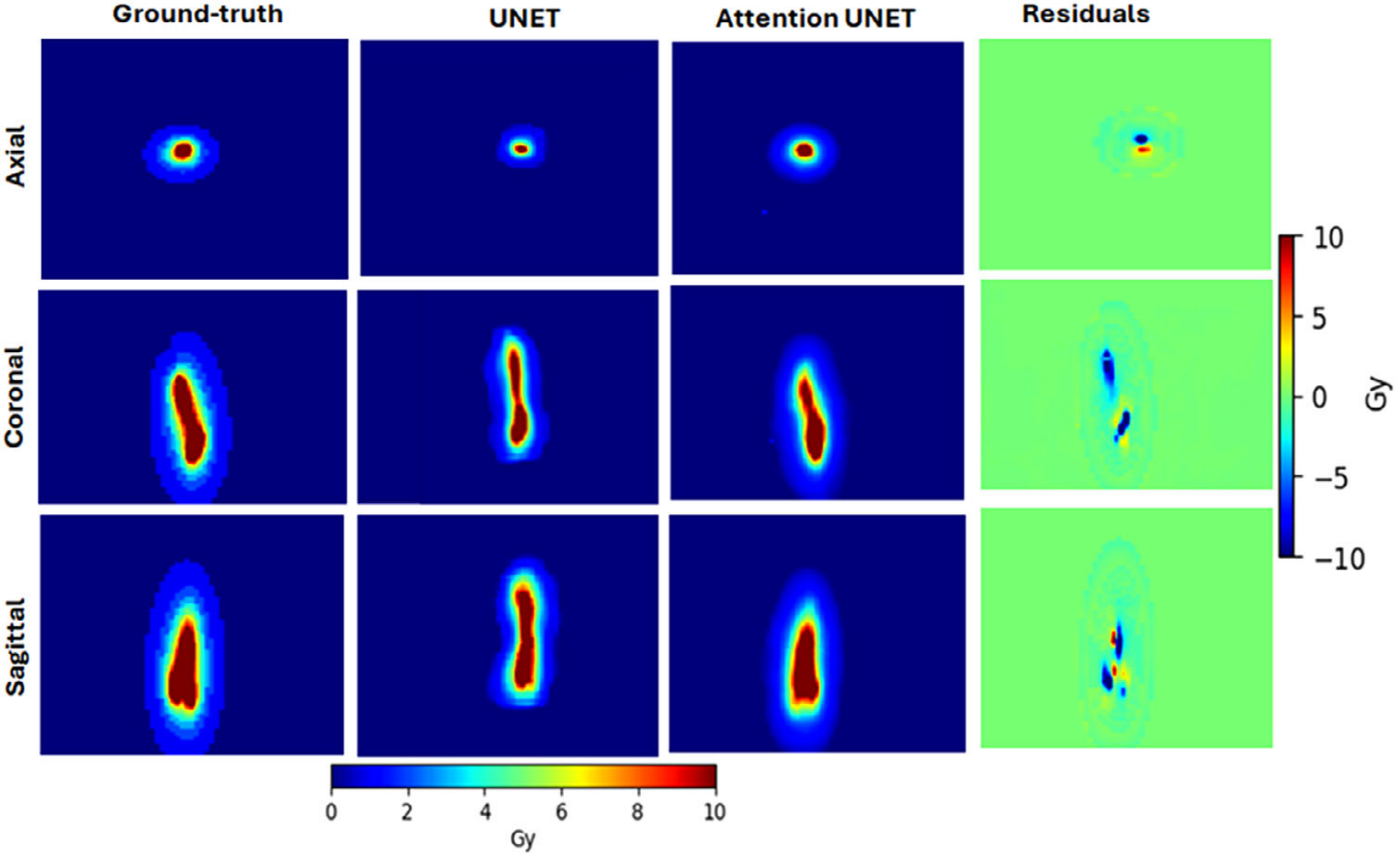
The predicted dose distribution (for UNET and attention‐UNET), ground‐truth dose distribution, and voxel‐wise difference map in axial, coronal, and sagittal planes for a sample patient in the test set. The horizontal color bar represents the dose for three planes, and the vertical color bar indicates the residual dose (the voxel‐wise dose difference between the attention‐UNET and the ground truth).

Figure 3 illustrates the predicted dose distributions in different planes (axial, coronal, and sagittal) alongside the ground‐truth dose distributions as an example from the test set. The shape of the predicted dose distributions closely resembled that of the ground‐truth dose distribution. As expected, significant dose differences were primarily concentrated near the treatment target due to the high‐dose gradient nature of BT.

The discrepancy between the predicted dose generated by the model and the ground‐truth was estimated using the MAE metric. The results of MAE values evaluated for the CTV_HR_, bladder, rectum, and sigmoid are reported in Table 1. The average MAE in the CTV_HR_ dose was 0.46 ± 11.71 Gy for attention UNET and 0.47 ± 9.16 Gy for UNET. For the bladder, rectum, and sigmoid, the values were 0.55 ± 0.67, 0.42 ± 0.46, and 0.31 ± 0.65 Gy for attention‐UNET, and 0.71 ± 1.54, 0.49 ± 1.34, 0.30 ± 3.76 Gy for UNET, respectively. The median absolute error (MdAE) in CTV_HR_ dose was 2.11 Gy for attention‐UNET and 2.84 Gy for UNET. Similarly, the MdAE values for the bladder, rectum, and sigmoid were 0.54, 0.50, and 0.47 Gy for attention‐UNET and 0.46, 0.40, and 0.32 Gy for UNET, respectively. Overall, the attention‐UNET showed a slight improvement compared to UNET. The difference, however, is not statistically significant and within the estimated standard deviation.

**TABLE 1:** Mean absolute error (MAE) and median absolute error (MdAE) for attention‐UNET and UNET (mean ± standard deviation).

	Attention‐UNET		UNET	
Structures	MAE (Gy)	MdAE (Gy)	MAE (Gy)	MdAE (Gy)
CTV_HR_	0.46 ± 11.71	2.11	0.47 ± 9.16	2.84
Bladder	0.55 ± 0.67	0.54	0.71 ± 1.54	0.46
Rectum	0.42 ± 0.46	0.50	0.49 ± 1.34	0.40
Sigmoid	0.31 ± 0.65	0.47	0.30 ± 3.76	0.32

Dose‐volume indices were calculated for the predicted and ground‐truth plans for the target and OARs. The MAEs, represented by the D_90_, V_100%_, V_150%_, and V_200%_ for the CTV_HR_, and D_2cc_ and D_max_ for the OARs, are shown in Table 2. The MAEs of predicting the D_90_, V_100%_, V_150%_, and V_200%_ in the CTV_HR_ were −0.45 ± 2.42 Gy (4.8%), 0.55 ± 9.42% (7.5%), 0.82 ± 4.21% (8.2%), and −0.80 ± 10.48% (10.3%), respectively. The MAEs for the D_2cc_ were 0.38 ± 1.19 Gy (9.1%) for the bladder, 0.43 ± 0.71 Gy (5.7%) for the rectum, and −0.47 ± 0.79 Gy (8.6%) for the sigmoid. Similarly, the values for the D_1cc_ were 0.09 ± 1.21 Gy (3.2%) for the bladder, 0.20 ± 0.95 Gy (9.9%) for the rectum, and −0.21 ± 0.59 Gy (8.1%) for the sigmoid.

**TABLE 2:** Mean absolute error between the predicted and clinical plan of quantitative dosimetric parameters for UNET and attention‐UNET.

		Dosimetric difference (Gy) [predicted—ground‐truth] % difference = [Dosimetric difference / ground‐truth]	
		Attention‐UNET	UNET
Structures	Metrics (Units)	MAE	MAE
CTV_HR_	^a^D_90_ (Gy)	−0.45 ± 2.42 (4.8 %) (*p* = 0.26)	0.43 ± 1.63 (4.2%) (*p* = 0.46)
	^b^V_100%_ (%)	0.55 ± 9.42 (7.5%) (*p* = 0.78)	0.52 ± 9.42 (8.9%) (*p* = 0.89)
	^c^V_150%_ (%)	0.82 ± 4.21 (8.2%) (*p* = 0.81)	0.84 ± 4.14 (9.6%) (*p* = 0.87)
	^d^V_200%_ (%)	−0.80 ± 10.48 (10.3%) (*p* = 0.36)	0.96 ± 14.48(12.96%) (*p* = 0.45)
Bladder	^e^D_2cc_ (Gy)	0.38 ± 1.19 (9.1%) (*p* = 0.50)	0.53 ± 0.74 (8.9%) (*p* = 0.23)
	^f^D_1cc_ (Gy)	0.09 ± 1.21 (3.2%) (*p* = 0.36)	0.42 ± 0.84 (5.2%) (*p* = 0.31)
	^g^D_max_ (Gy)	−0.42 ± 1.99 (6.3%) (*p* = 0.11)	0.45 ± 1.83 (8.0%) (*p* = 0.24)
Rectum	D_2cc_ (Gy)	0.43 ± 0.71 (5.7%) (*p* = 0.41)	0.59 ± 0.46 (6.2%) (*p* = 0.42)
	D_1cc_ (Gy)	0.20 ± 0.95 (9.9%) (*p* = 0.24)	0.21 ± 0.50 (10.5%) (*p* = 0.21)
	D_max_ (Gy)	0.04 ± 2.33 (2.8%) (*p* = 0.49)	0.39 ± 1.57 (6.5%) (*p* = 0.39)
Sigmoid	D_2cc_ (Gy)	−0.47 ± 0.79 (8.6%) (*p* = 0.30)	0.46 ± 0.60 (8.3%) (*p* = 0.11)
	D_1cc_ (Gy)	−0.21 ± 0.59 (8.1%) (*p* = 0.30)	0.52 ± 0.77 (8.9%) (*p* = 0.04)
	D_max_ (Gy)	−0.68 ± 1.94 (8.4%) (*p* = 0.13)	0.64 ± 1.45 (7.4%) (*p* = 0.64)

Results are reported in the form of mean ± standard deviation. The *p*‐values are also provided.

^a^ D_90_: Dose received by 90% of the volume.

^b^ V_100:_ % of volume receiving 100% of the prescription dose.

^c^ V_150:_ % of volume receiving 150% of the prescription dose.

^d^ V_200:_ % of volume receiving 200% of the prescription dose.

^e^ D_2cc_: Dose delivered to 2 cubic centimeter volume.

^f^ D_1cc_: Dose delivered to 1 cubic centimeter volume.

^g^ D_max_: Maximum dose received.

**FIGURE 4:**
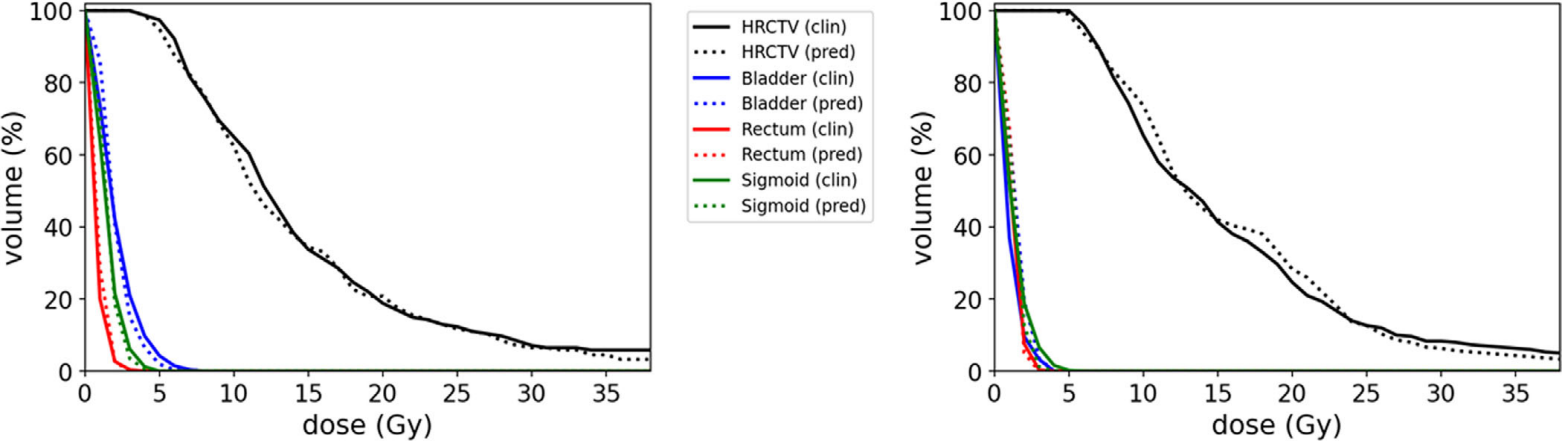
DVHs of a predicted plan (dashed lines) overlaid on DVHs of the ground‐truth plan (solid line*s*) of two sample patient plans in the test set with a prescription dose of 7 Gy and OAR cumulative doses below the limits of bladder D2cc < 90 Gy, rectum D2cc < 75 Gy, and sigmoid D2cc < 75 Gy in EQD2.

Figure 4 shows a DVH comparison between the predicted and ground truth plans for two samples in the test set, with a prescription dose of 7 Gy and the OAR cumulative doses below the limits of bladder D_2cc _< 90 Gy, rectum D_2cc _< 75 Gy, and sigmoid D_2cc _< 75 Gy in EQD2. The curves illustrate the CTV_HR_ and OAR structures of the two plans mostly coincide. It shows that the predicted DVHs of CTV_HR_ and OARs closely resemble ground‐truth DVHs.

These results indicate that the model holds significant promise in predicting useful dosimetric indices and enhances the workflow of BT treatment planning. Moreover, the model requires less than 5 s to generate a complete 3D dose distribution with dimensions of 64 × 64 × 64 voxels for any new patient's plan. This makes it suitable for near‐time applications and offers valuable support for clinical decision‐making.

## 4 DISCUSSION

BT enables precise delivery of the radiation dose directly to the target, which offers a more favorable therapeutic benefit than EBRT.^34^ Integrating an attention‐gating mechanism into a 3D UNET enhances efficiency and expedites model convergence by diminishing redundancies within the network. This model utilizes patient volumetric information, including CT images, structure set information, and their corresponding dose distributions as input.

To mitigate the likelihood of model overfitting and enhance its ability to generalize, a dropout strategy, data augmentation, and early stopping regularization were implemented. Therefore, the learning curve shown in Figure 2 demonstrated good‐fitting patterns during the training and validation. While achieving a high‐resolution dose distribution is a significant goal, it is extremely essential to weigh the trade‐off between computational time and memory requirements. In this study, we opted for a dimension of 64 × 64 × 64 (spatial resolution of 1 × 1 × 3 mm^3^) to gain a balance between computational efficiency and memory constraints. We believe that finer resolution could improve the prediction accuracy, specifically at the regions of high‐dose gradient; however, our computational memory does not have enough capacity to process larger data. To enhance the precision of the dose distribution, we integrated an attention‐gated module. This module is designated to employ additive self‐attention gates for adjusting the propagation of feature responses at various scales within the network. Network training of the attention‐gate 3D UNET took approximately 2 h for 250 epochs. The dose distribution prediction takes less than 5 seconds on NVIDIA Quadro RTX 6000 GPU. Figure 3 demonstrates that the attention‐gated 3D UNET successfully predicted the dose distribution almost identical to the ground‐truth. Even though we could not display the overlay of the predicted dose distributions on the anatomical structures (Figure 3), the authors believe that it is clinically crucial as a treatment plan is routinely evaluated quantitatively (DVH metrics) and qualitatively (iso‐dose lines overlaid on the anatomical structures).

The model achieved low MAE in CTV_HR_ (5.1%), as well as OARs, including the bladder (3.1%), rectum (4.0%), and sigmoid (3.2%), when the comparator was the attention‐gated 3D UNET predicted dose distribution. These results were also comparable to those published by Osman et al.^35^ method using the attention‐gated 3D UNET, (3.5% relative to prescription dose), to predict the dose distribution of head‐and‐neck cancer for EBRT. Moreover, the performance of the attention‐gated 3D UNET for predicting the dose for CTV_HR_ (MAE = 0.46 Gy), bladder (MAE = 0.55 Gy), rectum (MAE = 0.42 Gy), and sigmoid (MAE = 0.31 Gy) was comparable to Li et al.^36^ method using UNET (CTV_HR_: MAE = 0.34 Gy, bladder: MAE = 0.25 Gy, rectum: MAE = 0.25 Gy, and sigmoid: MAE = 0.22 Gy). The model tends to report a marginally high spread in CTV_HR_ as shown in Tables 1 and 2. This issue is because BT dose distribution diverges rapidly near the applicator due to the large dose gradients. As a result, the prediction errors in this high gradient and high‐dose regions could contribute disproportionately to a typical loss function of a neural network to reach the global minimum.

As shown in Figure 4, there was a good agreement between the predicted and ground‐truth plans for DVH analysis. The DVH metrics (D_90%_, V_100%_, V_150%_, V_200%,_ D_2cc_, D_1cc_, and D_max_) and dose distribution metrics obtained by our model were similar to the ground‐truth. V150% gives an indication of the hot spot, while V200% is clinically important to evaluate and ensure that the hot spot is within the applicator/gross tumor as much as possible. The MAE was −0.45 Gy for D_90_, 0.82% for V_150%_, and −0.80% for V_200%_. The mean absolute for V_100%_ was 0.55%, which is superior to Ming et al.^22^ method using 3D‐Deep CNN (1.2%). The MAE of the D_2cc_ index for bladder, rectum, and sigmoid within all the regions of interest was 0.38, 0.43, and −0.47 Gy, respectively. These results are almost comparable to those reported by Ming et al.^22^ (0.20, 0.25, and 0.11 Gy, respectively). Similarly, the MAE of the D_1cc_ index for bladder, rectum, and sigmoid was 0.09, 0.20, and −0.21 Gy, respectively, which is superior to Li et al.^36^ method using a squeeze and excitation attention‐Net (0.24 Gy for bladder and 0.33 Gy for rectum). The proposed attention‐gated 3D UNET showed a slight improvement in comparison to UNET. The reported results (Tables 1 and 2) of the predations were associated with high uncertainties, with larger deviations in target/OAR metrics when the predicted dose by Attention‐UNET is compared to the ground truth dose distributions. This could be due to the relatively small size of the test set, sharp dose gradient near the target, very high dose inside the applicator, and the slightly coarse spatial resolution of the data.

In clinical practice, creating an acceptable treatment plan often involves iterative planner adjustments. The goal is to achieve an optimal treatment outcome by maximizing the dose delivered to the target while minimizing the impact on OARs. However, this task is intricate due to conflicting objectives. Treatment plans are designed based on established guidelines that set dose limits. Nonetheless, these constraints do not guarantee an optimal dose distribution tailored to the unique anatomy and tumor location of each patient. They merely ensure that normal tissues remain within the specified dose limits. In addition, the treatment planning process map in BT is composed of high‐risk areas starting from treatment planning to treatment delivery where the patients are either under sedation or general anesthesia with applicators inserted. To expedite this process map and to generate the optimal plans within a time limit, it is necessary to develop a standardized and accurate dose prediction method. The proposed model in this study offers several advantages and potential uses. It could serve as a tool for quality assurance (QA) of the clinical treatment plan. Therefore, the planners can know in advance the feasibility of improving the dose distributions of the treatment plan. Moreover, the physicians can instantly visualize the 3D dose distributions and make necessary adjustments for the dose constraints of OARs. The planners could also take advantage of the predicted OARs DVHs from the dose distributions to define optimization objective function that may improve the quality and consistency of treatment plans and reduce planning time. Furthermore, the predicted 3D dose distributions could be used for the post‐optimization process to determine the dwell times of the source positions to generate a deliverable plan. To conclude, the BT treatment planning process encompasses several high‐risk stages from treatment planning to treatment delivery. This study aids in identifying and prioritizing these stages.

In addition to potential improvements in the planning process, this approach could enhance the standardization of the final treatment plans, not just the planning method. It also offers the possibility of being used as a QA tool for both the planning technique and the resulting plans. However, for it to serve as an effective QA too, the model would need to be retrained using only the highly optimal plans and future work could involve incorporating a method to detect and flag potential outliers or suboptimal plans automatically. This may include identification of poor plan quality (high doses to OARs or poor target coverage), conflicts in plan optimization (if inverse planning is used), uneven distribution of dwell times or overuse of channels, undesirably heterogeneous dose distributions (prone to inter/intra‐fraction motion errors), or high levels or hot spots within the dose distribution (V150‐V200).

In the future, we are considering using model‐based dose calculation algorithms (MBDCA) to calculate dose distributions. The dose map generated by BV‐TPS is calculated according to AAPM Task Group 43, which ignored the impact of tissue inhomogeneity and applicator heterogeneities.^5^ This issue becomes unavoidable when we expand this study in predicting dose distributions from direction‐modulated brachytherapy (DMBT) concept applicators, which is the next phase of our study.^37^

This study has some limitations. The major one is the relatively small dataset used in this study. The proposed model could report more accurate dose predictions if larger data were included in developing the model. Also, the MAE or MSE loss function could not adequately capture the dosimetric attributes in BT, particularly in regions with high dose and high dose gradients. Alternative loss functions such as gradient loss and Huber loss could improve the prediction accuracy. Further improvement and expansion will be carried out to improve the dose prediction accuracy.

## 5 CONCLUSION

The authors successfully generated 3D dose distributions in intracavitary HDR‐BT by integrating a 3D UNET and the attention‐gating DL mechanism, ensuring precise prediction of 3D dose distributions. This model has exhibited significant potential for clinical applications, enhancing the effectiveness of BT treatment planning workflow while ensuring consistent plan quality. The time required for prediction is in the order of seconds, allowing for the rapid generation of a complete 3D dose distribution, thus rendering it suitable for real‐time applications. The model can serve as a planning reference and be integrated into the plan optimization procedures for fully automated treatment planning processes, thereby streamlining the treatment planning workflow.

## AUTHOR CONTRIBUTIONS

Suman Gautam contributed to the conception, design, model development, drafting and revision of the manuscript, Alexander F.I. Osman contributed to model development and revision of manuscript, Dylan Richeson, Somayeh Gholami, Binod Manandhar, Sharmin Alam contributed to data preparation and revision of manuscript. William Y. Song contributed to conception and revision of manuscript. All authors contributed to manuscript revision and approved the submitted version.

## CONFLICT OF INTEREST STATEMENT

The authors declare no conflicts of interest.
